# Association Analysis of the Adenosine A1 Receptor Gene Polymorphisms in Patients with Methamphetamine Dependence/Psychosis

**DOI:** 10.2174/157015911795016958

**Published:** 2011-03

**Authors:** Hideaki Kobayashi, Hiroshi Ujike, Nakao Iwata, Toshiya Inada, Mitsuhiko Yamada, Yoshimoto Sekine, Naohisa Uchimura, Masaomi Iyo, Norio Ozaki, Masanari Itokawa, Ichiro Sora

**Affiliations:** 1 Department of Biological Psychiatry, Tohoku University Graduate School of Medicine, Sendai 980-8574, Japan; 2 Department of Neuropsychiatry, Okayama University Graduate School of Medicine, Dentistry and Pharmaceutical Sciences, Okayama 700-8558, Japan; 3 Department of Psychiatry, Fujita Health University School of Medicine, Aichi 470-1192, Japan; 4 Department of Psychiatry, Seiwa Hospital, Institute of Neuropsychiatry, Tokyo 162-0851, Japan; 5 Department of Psychogeriatrics, National Institute of Mental Health, National Center of Neurology and Psychiatry, Tokyo 187-8553, Japan; 6 Division of Medical Treatment & Rehabilitation, Center for Forensic Mental Health, Chiba University, Chiba 260-8670, Japan; 7 Department of Neuropsychiatry, Kurume University School of Medicine, Kurume 830-0011, Japan; 8 Department of Psychiatry, Graduate School of Medicine, Chiba University, Chiba 260-8670, Japan; 9 Department of Psychiatry, Nagoya University Graduate School of Medicine, Nagoya 466-8550, Japan; 10 Schizophrenia Research Project, Tokyo Institute of Psychiatry, Tokyo 156-8585, Japan; 11 Japanese Genetics Initiative for Drug Abuse (JGIDA), Japan

**Keywords:** Single nucleotide polymorphism, SNP, variation, human, Japanese, MAP, abuse, dopamine.

## Abstract

Several lines of evidence suggest that the dopaminergic nervous system contributes to methamphetamine (METH) dependence, and there is increasing evidence of antagonistic interactions between dopamine and adenosine receptors in METH abusers. We therefore hypothesized that variations in the A1 adenosine receptor (*ADORA1*) gene modify genetic susceptibility to METH dependence/psychosis. In this study, we identified 7 single nucleotide polymorphisms (SNPs) in exons and exon-intron boundaries of the *ADORA1* gene in a Japanese population. A total of 171 patients and 229 controls were used for an association analysis between these SNPs and METH dependence/psychosis. No significant differences were observed in either the genotypic or allelic frequencies between METH dependent/psychotic patients and controls. A global test of differentiation among samples based on haplotype frequencies showed no significant association. In the clinical feature analyses, no significant associations were observed among latency of psychosis, prognosis of psychosis, and spontaneous relapse. These results suggest that the *ADORA1* gene variants may make little or no contribution to vulnerability to METH dependence/psychosis.

## INTRODUCTION

Methamphetamine (METH) is a psychomotor stimulant with high liability for abuse, and METH abuse has become a very serious social problem in Japan [1]. Chronic METH abusers have been shown to have persistent dopaminergic deficits [2, 3]. Amphetamines are thought to produce their stimulant effects mainly *via* the dopaminergic system [4, 5], although other systems may also be involved. Dopamine D1 and D2 receptors form heterodimeric complexes with adenosine A1 and A2a receptors respectly, which modulate their responsiveness [6-9], suggesting that responses to amphetamines may also depend on adenosinergic function.

Several lines of evidence suggest that adenosine A1 receptors play a role in inhibiting the effects of METH. Adenosine receptor antagonists potentiate the effects of lower METH doses and substitute for the discriminative stimulus effects of METH [10, 11]. Adenosine receptor agonists protect against METH-induced neurotoxicity, and amphetamine-induced stereotypy and locomotor activity, and reduce the acquisition of conditioned place preference induced by amphetamine [12-15]. These results suggest that adenosine A1 receptors play important roles in the expression of METH-induced neurotoxicities and behaviors.

To date, however, there has been no association analysis between A1 adenosine receptor (*ADORA1*) gene variants and drug addiction. The purpose of this study was (1) to identify novel sequence variants in all coding exons as well as exonintron boundaries of the *ADORA1* gene in Japanese, and (2) to investigate whether these polymorphisms and/or haplotypes were associated with METH dependence/psychosis.

## MATERIALS AND METHODS

### Subjects

One-hundred seventy-one unrelated patients with METH dependence/psychosis (138 males and 33 females; mean age 37.5±12.0 years) meeting ICD-10-DCR criteria (F15.2 and F15.5) were used as case subjects; they were outpatients or inpatients of psychiatric hospitals. The 229 control subjects (119 males and 110 females; mean age 41.2±12.3 years) were mostly medical staff members who had neither personal nor familial history of drug dependence or psychotic disorders, as verified by a clinical interview. All subjects were Japanese, born and living in the northern Kyushu, Setouchi, Chukyo, Tokai, and Kanto regions. This study was approved by the ethical committees of each institute of the Japanese Genetics Initiative for Drug Abuse (JGIDA), and all subjects provided written informed consent for the use of their DNA samples for this research [16]. After informed consent was obtained, blood samples were drawn and genomic DNA was extracted by the phenol/chloroform method.

### Defining Variants of the *ADORA1* Gene

Initially, DNA samples from 16 METH dependent/psychotic patients were used to identify nucleotide variants within the *ADORA1* gene (GenBank accession no. AC105940). Exon numbers were based on the report by Ren and colleagues [17]. Exons 1A, 1B, 2, 3 and exon-intron boundaries were amplified by polymerase chain reaction (PCR) using a thermal cycler (Astec, Fukuoka, Japan), and the products were sequenced in both directions using BigDye terminators (Applied Biosystems, Foster City, CA) by an ABI Genetic analyzer 3100 (Applied Biosystems). The primer sequences used in this study are shown in Table **1**.

Genotyping of IVS1A+182 (rs56298433) was performed by PCR amplification using 2F-2R primers followed by restriction enzyme *Nla* III digestion. Genotyping of Exon2+363 (rs10920568) was performed by PCR amplification using 4F-4R primers followed by sequencing with the same primers. IVS2+35826 (rs5780149) was performed by PCR amplification using 5F-9R primers followed by sequencing with 5F and 5R primers. Genotyping of Exon3+937 (rs6427994), Exon3+987 (rs41264025), and Exon3+1064 (rs16851030) was performed by PCR amplification using 5F-9R primers followed by sequencing with 7F and 7R primers.

### Patient Subgroups

For the clinical category analysis, the patients were divided into two subgroups by three different clinical features. (A) Latency of psychosis from first METH intake: less than 3 years or more than 3 years. The course of METH psychosis varied among patients, with some patients showing psychosis sooner after the first METH intake, as previously reported [16, 18]. Because the median latency was 3 years, this time point was used as the cutoff in defining the two groups. (B) Duration of psychosis after the last METH intake: transient (<1 month) or prolonged (≧1 month). Some patients showed continuous psychotic symptoms even after METH discontinuation, as previously reported [16, 18]. Patients with the transient type showed a reduction of psychotic symptoms within one month after the discontinuation of METH consumption and the beginning of treatment with neuroleptics. Patients with the prolonged type showed a psychotic symptoms continued for more than one month even after the discontinuation of METH consumption and the beginning of neuroleptic treatment. (C) Spontaneous relapse: present or not. It has been well documented that once METH psychosis has developed, patients in the remission phase are liable to spontaneous relapse without reconsumption of METH [16, 18].

### Statistical Analysis

The Hardy-Weinberg equilibrium of genotypic frequencies in each SNP was tested by the chi-square test. The level of statistical significance was set at α= 0.05. The allelic and genotypic frequencies of the patient and control groups were compared using the chi-square test. Haplotype frequencies were calculated by the Arlequin program available from http://anthropologie.unige.ch/arlequin [19]. Locus by locus linkage disequilibrium (LD) was evaluated by D’ and r^2^, which were calculated by the haplotype frequencies using the appropriate formula in the Excel program. A global test of differentiation among samples based on haplotype frequencies was also performed by the Arlequin program.

## RESULTS

### Analysis of the *ADORA1* Gene Variants

To identify polymorphisms in the *ADORA1* gene, exons 1A, 1B, 2, and 3, and exon-intron boundaries were analyzed using genomic DNA from Japanese METH dependent/psychotic subjects. Seven SNPs were identified (Table **2**). Five out of seven of these SNPs were previously reported by Deckert [20]. In the two SNPs, the frequencies of the minor alleles differed between our patients and those of Deckert. In the Exon2+363 (rs10920568) SNP, the G allele was present in 15.5% of our Japanese controls (Table **3**) and 36.9% of the German controls [20]. In the Exon3+1064 (rs16851030) SNP, the T allele was present in 35.8% of our Japanese controls and 1.2% of the German controls [20]. These differences were suggested to be related to the difference in ethnicity between the two cohorts. One SNP, Exon2+363 (rs10920568), was a synonymous mutation (Ala to Ala) (Table **2**). All the other SNPs were located either in the introns or an untranslated region in the exon 3. Two SNPs (Exon3+937 (rs6427994) and Exon3+1454 (rs11315020)) were in linkage disequilibrium (LD) in the sense that the genotypic patterns of the 16 samples examined were the same, representing Exon3+937 (rs6427994) for these two SNPs. IVS1A+182 (rs56298433), Exon2+363 (rs10920568), IVS2+35826 (rs5780149), Exon3+937 (rs6427994), Exon3+ 987 (rs41264025), and Exon3+1064 (rs16851030) were chosen for further analysis.

### Relationship Between the *ADORA1* Gene SNPs and METH Dependence/Psychosis

Association analyses between these SNPs in the *ADORA1* gene and METH dependence/psychosis were performed using DNA samples from 171 METH dependent/psychotic subjects and 214 control subjects (Table **3**). Among them, the genotypes of five control samples and three METH samples could not be determined at IVS1A+182 (rs56298433). The genotypic frequencies in these SNPs were within the Hardy-Weinberg expectations. No significant differences of the genotypic and allelic distributions of these SNPs in these samples were observed. As the minor allele frequencies of two SNPs, IVS1A+182 (rs56298433) and Exon3+987 (rs41264025), were less than 5%, another four SNPs, Exon 2+363 (rs10920568), IVS2+35826 (rs5780149), Exon3+937 (rs6427994), and Exon3+1064 (rs16851030), were used for further analyses.

A global test of differentiation among samples based on haplotype frequencies was performed using the Arlequin program, but no significant association with METH dependence/psychosis was observed (P=0.590). Haplotype frequencies were estimated by the Arlequin program, and locus by locus LD was calculated by using the appropriate formula in the Excel program. Most of the SNPs in exon 2 and exon 3 were in LD, suggesting that the locus from exon 2 to exon 3 was in a LD block (Table **4**). 

Subcategory analyses were conducted on the clinical parameters (latency of psychosis, prognosis of psychosis, and spontaneous relapse) (Table **5**). Significant differences were observed in the shorter latency of psychosis (P=0.025) at Exon3+937 (rs6427994). However, this significance disappeared after Bonferroni correction by the sub-group numbers, two (P < 0.025).

## DISCUSSION

We analyzed the *ADORA1* gene variations in a Japanese population and found seven SNPs in exons and exon-intron boundaries. However, no significant associations were observed between these SNPs and METH dependence/psychosis in the genotypic, allelic, haplotypic or clinically subcategorized analyses.

This is the first association analysis between *ADORA1* gene variants and drug addiction. We failed to find associations between the *ADORA1* gene SNPs and METH dependence/psychosis. While the significant difference (P=0.025) in the shorter latency of psychosis at Exon3+937 (rs6427994) disappeared after Bonferroni correction, this may have been due to the sample size, and thus further analysis with a larger sample is warranted.

The variants we found were one synonymous SNP, two intron SNPs and four exon SNPs in the untranslated region. These SNPs are unlikely to affect receptor function because they are not non-synonymous SNPs or promoter SNPs. Because several animal studies have suggested a modulatory role of adenosine receptors for dopamine systems, it remains possible that another region in the *ADORA1* gene, such as a promoter region or intron regions, contributes to the alteration of *ADORA1* gene function.

	Although a few association analyses of the *ADORA1* gene and psychiatric diseases have been performed, no significant association has been reported between *ADORA1* variants and bipolar affective disorder or panic disorder [20, 21]. As caffeine is a nonselective adenosine receptor antagonist, the association between the psychoactive effects of caffeine and gene variants of adenosine receptors have also been studied. However, the anxiogenic response to an acute dose of caffeine in healthy, infrequent caffeine users was not associated with *ADORA1* gene polymorphism [22]. Interindividual variation in the anxiety response to amphetamine has also been studied in healthy volunteers, but no association was observed with *ADORA1* gene variants [23]. These results suggest that the *ADORA1* gene variations have little effect on psychiatric symptoms and/or personality traits.

In conclusion, our data suggest that the *ADORA1* gene variants may not play a major role in the development of METH dependence/psychosis.

## Figures and Tables

**Table 1:** Primers Used in this Study

Exon	Forward		Reverse	
Exon1A	1F:	TGG ACT GGA TGC CTT ATG GCT TAG	1R:	GGC GCA GGA GCT GAG TGA CAA TCG
	2F:	TCT CAC CCA GTA TCA CTT CCT TTG	2R:	ATC ACA TGG TAC GGC AGA GAC TCA
Exon1B	3F:	AAT AGG GAG AAA CGC CCC AGC CTT	3R:	AAG CAC CTG TGT GGT CAG GGA AGC
Exon2	4F:	GGT AGG AGC TGC ATG TGA CAA GTG	4R:	GCA GAG TGA GGA CTG GAG CAC GAT
Exon3	5F:	GGC TGT CAT GAA GCA ATG ATG AGA	5R:	CCA GCG ACT TGG CGA TCT TCA GCT
	6F:	TCT ACC TGG AGG TCT TCT ACC TAA	6R:	CCC TGA AGC TCT GGA CTG CTC ATG
	7F:	GTG GTC CCT CCA CTA GGA GTT AAC	7R:	ACA GGT AAT TAC ACT CCA AGG CTC
	8F:	CTG ATA TTT GCT GGA GTG CTG GCT	8R:	ACA CCT GCA ACA GAG CTT CCA AAG
	9F:	CCT TGC TGT CAT GTG AAT CCC TCA	9R:	CAA GAG GAA GAT GCC AAT GGG AGA

**Table 2:** *ADORA1* Gene Variants Found in the Japanese Population

Location	Variants	rs#	SNP Name	Function
IVS1A+182	G/T	rs56298433		intron
Exon2+363	T/G	rs10920568	805T/G	synonymous (Ala->Ala)
IVS2+35826	T4/T5	rs5780149		intron
Exon3+937	A/C	rs6427994	1777C/A	untranslated
Exon3+987	C/T	rs41264025	=1827C/T	untranslated
Exon3+1064	C/T	rs16851030	1904C/T	untranslated
Exon3+1454	T/del	rs11315020	2294insT	untranslated

The nucleotide sequence of the *ADORA1* gene was referenecd to the NCBI nucleotide database under accession number AC105940. Exon numbers were based on the report by Ren and colleagues [17]. The column labelled rs# shows SNP numbers from the NCBI SNP database. The data in the column labelled SNP name are from the report by Deckert [20].

**Table 3:** Genotypic and Allelic Distribution of the *ADORA1* Gene SNPs in the METH Subjects and the Controls

SNP	Group	N	Genotype (%)			P	Allele (%)		P
IVS1A+182 (rs56298433)			G	G/T	T		G	T	
	Control	224	222 (99.1%)	2 (0.9%)	0 (0.0%)	0.961	446 (99.6%)	2 (0.4%)	0.823
	METH	168	166 (98.8%)	2 (1.2%)	0 (0.0%)		334 (99.4%)	2 (0.6%)	
Exon2+363 (rs10920568)			T	T/G	G		T	G	
	Control	229	162 (70.7%)	63 (27.5%)	4 (1.7%)	0.333	387 (84.5%)	71 (15.5%)	0.233
	METH	171	132 (77.2%)	36 (21.1%)	3 (1.8%)		300 (87.7%)	42 (12.3%)	
IVS2+35826 (rs5780149)			T4	T4/T5	T5		T4	T5	
	Control	229	150 (65.5%)	69 (30.1%)	10 (4.4%)	0.887	369 (80.6%)	89 (19.4%)	0.708
	METH	171	108 (63.2%)	55 (32.2%)	8 (4.7%)		271 (79.2%)	71 (20.8%)	
Exon3+937 (rs6427994)			A	A/C	C		A	C	
	Control	229	2 (0.9%)	46 (20.1%)	181 (79.0%)	0.248	50 (10.9%)	408 (89.1%)	0.222
	METH	171	5 (2.9%)	38 (22.2%)	128 (74.9%)		48 (14.0%)	294 (86.0%)	
Exon3+987 (rs41264025)			C	C/T	T		C	T	
	Control	229	215 (93.9%)	14 (6.1%)	0 (0.0%)	0.937	444 (96.9%)	14 (3.1%)	0.888
	METH	171	162 (94.7%)	9 (5.3%)	0 (0.0%)		333 (97.4%)	9 (2.6%)	
Exon3+1064 (rs16851030)			C	C/T	T		C	T	
	Control	229	89 (38.9%)	116 (50.7%)	24 (10.5%)	0.071	294 (64.2%)	164 (35.8%)	0.572
	METH	171	80 (46.8%)	67 (39.2%)	24 (14.0%)		227 (66.4%)	115 (33.6%)	

N: number of samples.

P: Significance values between the METH subjects and the controls.

**Table 4:**
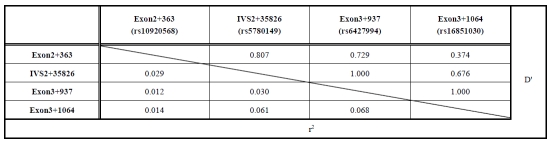
Linkage Disequilibrium Mapping of the *ADORA1* Gene

D' and r^2^ values for Controls are shown in the upper right and lower left, respectively.

**Table 5:** Genotypic Distribution of the *ADORA1* Gene SNPs in Subcategorized METH Subjects

		SNP	Exon2+363 (rs10920568)				IVS2+35826 (rs5780149)				Exon3+937 (rs6427994)				Exon3+1064 (rs16851030)			
		Genotype	T	T/G	G		T4	T4/T5	T5		A	A/C	C		C	C/T	T	
Group		N				P				P				P				P
Control		229	162	63	4		150	69	10		2	46	181		89	116	24	
METH	Latency of Psychosis																	
	<3 years	67	48	16	3	0.387	46	17	4	0.684	4	10	53	0.025	30	26	11	0.173
	≧3 years	71	56	15	0	0.275	40	29	2	0.229	0	22	49	0.124	35	28	8	0.237
	Prognosis of Psychosis																	
	Transient (<1 month)	91	70	19	2	0.465	59	29	3	0.883	3	22	66	0.190	42	37	12	0.269
	Prolonged (≧1 month)	56	41	14	1	0.932	33	20	3	0.654	1	11	44	0.835	27	21	8	0.205
	Spontaneous Relapse																	
	Not present	104	81	22	1	0.381	64	34	6	0.733	4	25	75	0.107	52	39	13	0.081
	Present	60	45	13	2	0.519	39	19	2	0.923	1	11	48	0.831	25	24	11	0.163

N: number of samples.

P: Significance values between the METH subjects and the controls.
